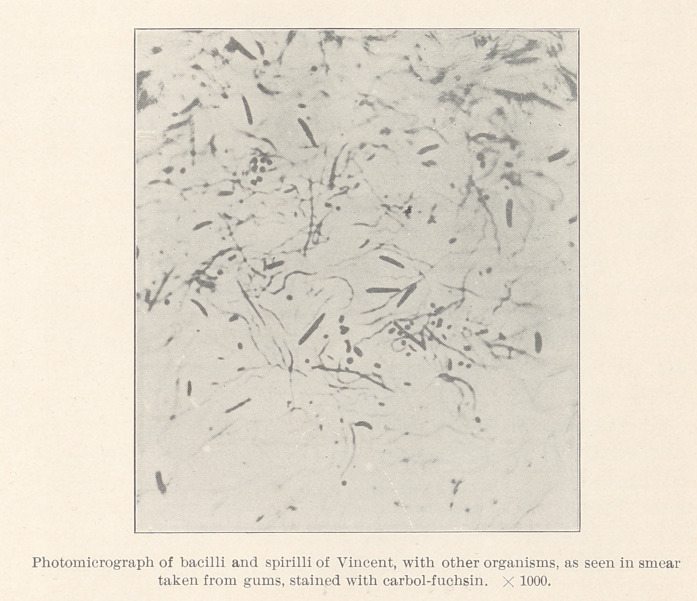# Report of a Case of Vincent’s Angina and Stomatitis

**Published:** 1904-11

**Authors:** G. C. Crandall

**Affiliations:** St. Louis


### REPORT OF A CASE OF VINCENT'S ANGINA AND STOMATITIS.[1]

BY G. C. CRANDALL, B.S., M.D., ST. LOUIS.

This infection of the throat and mouth, as described by those who have reported cases, is characterized by a membranous, ulcerative process, quite painful, but with slight systemic reaction—the lesions, especially of the mouth, usually healing slowly; the secretion, pseudo-membrane, and tissue beneath containing a fusiform bacillus associated, as a rule, with a spirillum.

As comparatively few cases of Vincent's angina have been reported in this country, the following case will be of interest:

*History.*—Patient, male, single, twenty-three years old, medical student, family history good, always having been well except for an attack of measles and of typhoid fever some years ago. No

---

[1] Read at the annual session of the American Medical Association, Section on Stomatology, Atlantic City, June 7 to 10, 1904.

venereal disease. He had never suffered from sore throat nor sore mouth of any kind, and his teeth were unusually good.

The first indication of the disease which he observed appeared one morning at breakfast, when he noticed that swallowing hot coffee caused some pain in the region of the left tonsil. Looking at his throat he found it somewhat congested on the left side. During the following day it became gradually worse, so that the mere act of deglutition was very painful, much more, however, when swallowing anything hot; the tonsil, soft palate, and uvula becoming more congested. The second day a small diphtheritic spot was observed on the upper anterior border of the left tonsil, and the pain increased somewhat.

The spot was about one-fourth inch in diameter, and did not enlarge much during the six days it was present. It was covered by a grayish-white, friable pseudo-membrane, which could be easily removed, leaving a slightly depressed bleeding surface, over which membrane would again form in a few hours.

The fourth day of the disease he had a dentist clean his teeth, and the following day the disease appeared along the margin of the gums and between the teeth, the gums rapidly receding from the teeth, and the infection extended in places over the gums to the buccal surface, especially about the last molar teeth. Wherever the infection extended it had the appearance of the primary spot on the tonsil,—ulceration, accumulation of pseudo-membrane, congestion of surrounding mucous membrane, bleeding of the ulcerative surface when disturbed, and pain. The bleeding of the gums was very annoying, and with the pain prevented him from eating anything which it was necessary to masticate. With the extension of the infection to the gums, the breath became very foul, due to decomposing blood and membrane about and between the teeth. This unpleasant symptom continued to some extent until the disease entirely disappeared.

During the early part of the attack there was a slight increase in salivary secretion, but of no consequence. There was some swelling of the lymphatic glands near the angle of the jaw on the side where the infection first appeared; later there was slight swelling and tenderness of the lymphatics of the submaxillary region after the gums were invaded.

Throughout the course of the attack there were only slight constitutional symptoms; temperature was raised one-half to one

Photomicrograph of bacilli and spirilli of Vincent, with other organisms, as seen in smear taken from gums, stained with carbol-fuchsin. $\times$ 1000.

degree during first few days, after which it was normal. The patient became somewhat debilitated because of his inability to take the usual amount of food, but continued attending his college work without missing a day. He drank liquids, and ate only bland soft food neither hot nor cold.

*Treatment.*—On the third day the patient began treatment, applying a ten per cent. silver solution without apparent effect. On the fourth day the spot of the tonsil was touched with pure carbolic acid, followed by a gargle which consisted of 1 to 1000 bichloride in two per cent. carbolic solution. This relieved the throat at once, but had little effect on the infection of the gums, which later was relieved by chlorate of potash in solution, and better in the form of tablets, which the patient dissolved in the mouth frequently, expectorating the saliva. The tablets were used to the end of the attack. The throat symptoms cleared up in a week, but the lesions about the gums resisted treatment much longer, showing a tendency to recur, apparently because of the infection between and about the teeth which was so inaccessible to the local remedies used. While the throat was well in a week, the gums showed traces of the disease for six weeks.

*Bacteriology.*—A smear was made from the tonsil on the fourth day, first drying the spot with cotton to remove the mucus from the surface. This showed the bacillus of Vincent and a spirillum, the latter appearing identical with the *Spirochœta dentium* (Cohn), which is common in the mouth. Both organisms were abundant, with very few other germs present. Smears taken from the margin of the gums showed both organisms, but with numerous other organisms from the decomposing material about the teeth.

The organisms stained readily with carbolic fuchsin, also with gentian violet, and with Loeffler's methylene blue. The bacillus took the stains, as a rule, much better than the spirillum, although the latter took the gentian violet fairly well.

Efforts to make cultures of the organisms on the common media, gelatin agar, and blood serum were all negative.

The bacilli were distinctly fusiform, averaging large, but varying in length from eight to twelve microns, and in thickness from one-half to one micron. The spirilli were thirty-six to forty microns long, and of quite uniform thickness, about one-third micron (see illustration).

The organisms were found abundant during the first few days of the disease; later only a few could be found.

In this case the disease was at first confined to the throat, but was quickly and thoroughly inoculated into the gums by the irritation incident to cleaning the teeth.

The dentist was not aware of the infectious process in the throat; however, this case illustrates the necessity of caution on the part of the dentist in so simple a procedure as cleaning the teeth when any acute infectious process exists about the throat or mouth; at most, then, only the teeth and not the gums should be disturbed; every precaution should be taken to avoid irritation of the mucous membrane, since the slightest abrasion is inoculated with the infected secretion.

When we have an acute infectious process of the throat or mouth which has a tendency to spread, it would be well to confine the diet of the patient to bland liquids and soft food requiring no mastication, thus avoiding, so far as possible, all irritation of the mucous membrane.

So far as known, no other cases developed, although the patient was associating with other students constantly, avoiding, however, using any common drinking-cup.

Briefly reviewing the literature, we find that in 1896 Vincent[1] reported a form of ulcerative angina due to these organisms. In 1897 Bernheim[2] reported a series of thirty cases which conform in general to this disease, although he did not feel certain that the fusiform bacilli and spirilli found were the cause. Vincent[3] again, in 1898, reported fourteen cases. In 1901 Nicolet[4] and Morotte described the morphology of the organism. Mayer[5] in 1902 reported a typical case, with clinical data. In 1903[6] Fisher reported two typical cases, with description of organisms and illustrations. Hess[7] in 1903 reported two forms of the disease,—the croupous form, due to the fusiform bacilli, and the diphtheritic form, in which both the bacilli and the spirilli are present. In 1903 Anchi[8]

---

[1] Annales de l'Institute Pasteur, 1896.

[2] Deutsche med. Woch., 1897.

[3] Bull. de la Soc. des Hôpitaux, March, 1898.

[4] Revue de Médecin, April 10, 1901.

[5] Jour. Am. Med. Sci., 1902, p. 187.

[6] Ibid., 1903, p. 438.

[7] Deutsche med. Woch., vol. xxix., No. 42.

[8] Gaz. Hebd. d. Sci. Méd. de Bordeaux, 1903, vol. xxiv. p. 555.

called attention to the possibility of considerable tissue destruction incident to the disease. In 1903 Tarruella [1] discussed the clinical and bacteriologic features of what he terms the ulcerative-necrotic angina of Vincent. In 1903 Conrad [2] reviewed the literature to date quite thoroughly and gave some clinical reports.

Most of the observers emphasize the tendency of the disease to run a protracted course, especially when the gums are affected.

The differential diagnosis will come, as a rule, within three diseases,—syphilis, diphtheria, and Vincent's angina,—which can usually be readily cleared up by the history of the attack and a microscopic examination of the secretion from the ulcerated surface.

### DISCUSSION.

*Dr. Vida A. Latham,* Rogers Park, Chicago.—I had the good fortune to see this specimen, which, I understand, is only the third ever reported in America. From a dental or stomatologic point of view it is of value, showing that dentists must recognize this disease. I had one case some time ago, but it was never recorded, as I was not sure at the time what it was. The patient's lips became almost black from the disease, and in consequence it was called gangrenous stomatitis. There was considerable pain and great nervous prostration. The only way of identifying the disease is by the microscopic examination.

*Dr. E. C. Briggs,* Boston.—I think I must have had a similar case, but no microscopic examination was made. When I first saw the case I thought it must be syphilis. There was excessive ulceration of the mucous membrane with severe pain. The patient was a man of character and courage, and I did not feel that he was exaggerating when he told me how intolerable his days and nights were. At another time I shall have a microscopic examination made for diagnosis. The case cleared up after a while, during which time I treated him vigorously.

*Dr. Latham.*—I would suggest in these cases the use of orthoform tablets for pain on deglutition.

---

[1] Rev. de Med. y Cirug. Barcel., 1903, vol. xvii. p. 180.

[2] Arch. f. Laryngol. u. Rhinol., Berlin, 1903, vol. xiv. 525.

## Figures and Tables

**Figure f1:**